# Quantifying Physical Activity in Toddlers with Down Syndrome with Wearable Sensors During a Home-Based Mobility Intervention

**DOI:** 10.3390/s26196002

**Published:** 2026-09-22

**Authors:** Varsha Athreya, Simoni Mishra, Ashley N. Collimore-Doherty, Ellen N. Sutter, Jana M. Iverson

**Affiliations:** 1 Department of Computer Engineering, Boston University, Boston, MA 02215, USA; vathreya@bu.edu; 2 Department of Biomedical Engineering, Boston University, Boston, MA 02215, USA; simonim@bu.edu; 3 Department of Physical Therapy, Boston University, Boston, MA 02215, USA; acollimo@bu.edu

**Keywords:** wearable sensor, accelerometer, physical activity, toddler, Down syndrome, intervention

## Abstract

**Highlights:**

**What are the main findings?**

- This study identified an accelerometry cutpoint for distinguishing moderate-to-vigorous physical activity from sedentary-to-light physical activity in toddlers with Down syndrome during an at-home mobility intervention.
- The group-derived cutpoint performed similarly to individually derived cutpoints in classification accuracy.

**What is the implications of the main finding?**

- Wearable accelerometers may be a feasible and practical approach to quantify physical activity during home-based mobility interventions.

**Abstract:**

Background: Quantifying movement during early mobility interventions is important for evaluating intervention participation and progression in toddlers with Down syndrome (DS), but behavioral video coding is time and labor-intensive. This study established and evaluated an activity count cutpoint for distinguishing moderate-to-vigorous physical activity (MVPA) and sedentary-to-light physical activity (SLPA) in toddlers with DS during a home-based mobility intervention using a wearable accelerometer integrated into a body-weight support harness system. Methods: Video recordings behaviorally coded for MVPA and SLPA were synchronized with accelerometer activity counts binned in 5 s epochs. Receiver operating characteristic curve analysis was used to determine both group-derived and participant-specific activity count cutpoints. Results: The optimal group-derived cutpoint was 141 activity counts per 5 s, demonstrating strong classification performance (AUC = 0.903), with 94.4% sensitivity, 70.2% specificity, and an overall classification accuracy of 79.2%. Participant-specific cutpoints resulted in only minimal improvements in classification performance. Conclusions: These findings suggest that using individualized cutpoints may not be necessary for classifying MVPA in toddlers with DS during this intervention, though replication in a larger sample is warranted. A standardized activity count cutpoint may provide a practical approach for objectively monitoring activity during home-based mobility interventions.

## 1. Introduction

Independent movement and exploration in infancy have far-reaching effects on overall development. Early motor milestones are not isolated physical achievements, but rather part of a developmental cascade where self-initiated movements are linked to the emergence of cognitive and language skills [1]. Infants and toddlers with Down Syndrome (DS) exhibit delays in the achievement of independent mobility [2,3] due to a combination of factors such as hypotonia, ligamentous laxity, and postural control impairments [2,4,5]. Such delays can limit opportunities for environmental exploration, which may in turn delay subsequent developmental advances across domains. Thus, interventions that support mobility are an important component of physical therapy for young children with DS and a focus of current DS rehabilitation research [6,7].

Growing evidence suggests that home-based mobility interventions, including treadmill training and body-weight support systems, can improve motor development in toddlers with DS [8,9,10]. These interventions allow toddlers to practice motor skills at home, which enables high-dosage practice, a key principle of motor learning [11,12]. Open-area body-weight support systems also allow toddlers to simultaneously explore their environment, experience different body positions, and interact with objects and people. However, assessing adherence rates and quantifying infant motor practice during these interventions is a challenge [8,13,14]. Evaluations of caregiver-delivered, at-home interventions often rely heavily upon caregiver reports (e.g., daily log), which can be subjective [15] and typically cannot provide a sufficiently granular record of the toddler’s activities during the intervention period. Behavioral coding from video recordings offers a precise record of children’s activity during an intervention session [8,13]; however, this method is time- and resource-intensive, and the presence of cameras or researchers may alter the natural behavior of children and caregivers [14]. Furthermore, video recording may not be practical during an in-home intervention delivered over weeks or months. Objective ways to characterize child movement are needed to evaluate how often and how much toddlers are practicing motor skills during at-home interventions and to track progress over time.

Wearable sensors offer a non-invasive way to monitor dosage and fidelity of home-based interventions in clinical populations [16,17,18], and quantify child movement and activity level during play [19,20]. By converting raw acceleration into a single value of intensity (activity counts), movement can be classified as sedentary, light, moderate, or vigorous physical activity using “cutpoints” [19]. Prior work has used wearable sensors to quantify physical activity in pediatric populations of all ages with and without developmental disabilities [21,22,23,24]. However, methodological differences in epoch length, sensor placement, and cutpoint criteria limit the generalizability of established physical activity cutpoints [24].

In addition, these studies have not evaluated cutpoint criteria for infants or toddlers with DS. Cutpoints established for typically developing (TD) toddlers may not accurately identify activity levels in toddlers with DS, who generally demonstrate lower levels of physical activity, may move more slowly [25,26], and adopt different postural and movement strategies relative to TD peers [5,27]. Furthermore, while group-level cutpoints are commonly used for TD toddlers [24], the heterogeneity in movement strategies across toddlers with DS may be a limitation to establishing a single group cutpoint. If a group cutpoint cannot be established with sufficient accuracy, individual cutpoints could be an alternate strategy for activity classification.

The purpose of this study was to establish the feasibility of using a single wearable sensor worn on the torso to classify and quantify physical activity during an at-home, play-based body-weight support intervention with toddlers with DS. We identified and tested group-level and individual-level physical activity cutpoints during a single intervention session, using behavioral coding of activity from video as a reference. We hypothesized that individual-level cutpoints would significantly improve accuracy and precision compared to a group-level cutpoint.

## 2. Materials and Methods

### 2.1. Participants

Participants were four pre-walking toddlers with DS recruited as part of a larger study investigating the feasibility and acceptability of a body-weight support system to enhance mobility (NCT06219863). See Table 1 for participant demographics. All procedures were approved by the Boston University Institutional Review Board, and caregivers provided informed consent for their child prior to participation.

### 2.2. Data Collection

Participants were video-recorded during a 15-min play session with a caregiver in their homes. During the play session, participants used the Portable Mobility Aid for Children (PUMA, Enliten LLC, Newark, DE, USA), a body-weight support harness system, to facilitate upright postures and movement (Figure 1). The PUMA was intentionally set up in a play-enriched environment, with furniture and foam climbing blocks positioned to encourage upright postures and movement. Otherwise, the play session was unstructured; caregivers and toddlers were asked to engage in play activities of their choosing (see [28] for more detail). Children were connected to the PUMA system via a cloth onesie. An inertial sensor (Opal sensor, APDM, Inc., Portland, OR, USA) was attached to the back of the onesie at the level of mid-torso. This placement was chosen to capture torso movements rather than minor leg and arm movements. Sensor data were recorded at 128 Hz for participants 2–4 in line with prior accelerometry work [20,29]. Data were recorded at 100 Hz for participant 1 due to setup error, but this was determined to be an acceptable frequency for measuring child movement. Video was recorded at 30 Hz. The video and sensor data were synchronized by tapping the sensor three times in view of the camera at the beginning of the session. These movements were then identified in the raw acceleration data and video data.

### 2.3. Data Processing

From each 15-min play session, a five-minute segment was selected from the middle of the session, when toddlers exhibited variable postures and movements. These segments were behaviorally coded using DataVyu (Version 1.5.3; datavyu.org), an open-source software program for manually annotating videos. Videos were coded in a binary manner (motion/no-motion) based on a predefined manual (Supplementary Material S1). “Motion” was defined as torso movement (e.g., rotation) visually estimated by the coder to be approximately 45 degrees or greater, transitions between postures (e.g., sit to stand), or locomotion (e.g., crawling, walking). Isolated leg or arm movement was not considered to be motion.

Toddler motion was analyzed in one-second intervals where each second was considered “motion” if the toddler was moving according to the criteria for more than half of the interval (e.g., >0.5 s). Intervals not classified as “motion” were classified as “no motion.” The one-second intervals were then grouped into five-second epochs where an epoch was classified as moderate-to-vigorous physical activity (MVPA) if three or more one-second intervals were classified as “motion”. Epochs were classified as sedentary-to-light physical activity (SLPA) if three or more one-second intervals were classified as “no-motion.” Five-second epochs were chosen based on previous work recommending an epoch length of less than 15 s for infant movement [24,30] to capture shorter movement bouts. Behavioral coding was conducted independently by two researchers (VA, SM) and discrepancies between the epoch classifications were discussed and resolved by consensus.

Raw triaxial accelerometer data were processed and analyzed using custom MATLAB scripts (Version R2024b; The Math Works Inc., Natick, MA, USA). Signal processing of acceleration followed the method outlined in Neishabouri et al. (2022) to emulate the computations done by ActiGraph (now Ametris) sensors to calculate activity counts [29] (Figure 2). Briefly, the linearized acceleration data was downsampled to 30 Hz, passed through a 7th-order infinite impulse response filter, rescaled by 17.13, rectified, thresholded between four and 128 m/s^2^, and downsampled again to 10 Hz. The preprocessed acceleration output was then grouped and summed across five-second epochs, providing activity counts [29].

### 2.4. Establishing Cutpoints

To distinguish between MVPA and SLPA, we determined an activity count cutpoint. Physical activity was categorized into either MVPA or SLPA rather than one of sedentary, light, or moderate-to-vigorous, since previous research suggests more consistency in identifying MVPA compared to the other two categories [30]. Epochs with an activity count value above the cutpoint were categorized as MVPA, and epochs with an activity count value below the cutpoint were considered SLPA. We took two approaches to determine a cutpoint for our sample: determining a DS group cutpoint from the combined data of the four participants, and individual cutpoints for each participant.

Separate receiver operating characteristic (ROC) curves were plotted using the *perfcurve()* function in MATLAB (Version R2024b) with (1) concatenated data from all four participants and (2) data from each individual participant. The group and individual cutpoints were identified as the activity count value that maximized true positives and minimized false positives (i.e., Youden Index).

### 2.5. Evaluation

We used the area under curve (AUC) of the ROC curve to evaluate how well activity count measures discriminated MVPA and SLPA. AUC values of greater than or equal to 0.90 were considered excellent, between 0.80 and 0.89 good, between 0.70 and 0.79 fair, and less than 0.70 poor [30]. We also calculated sensitivity (number of true positive epochs divided by the total number of positive frames) and specificity (number of true negative frames divided by the total number of negative frames) of the selected cutpoint. A sensitivity and specificity of 0.7–0.79 was considered “acceptable” and greater than 0.8 was considered “good” [20]. Lastly, we calculated the overall percent agreement between the behavioral coding classification and the activity count classification as the number of epochs that agreed divided by the total number of epochs; both the group and individual cutpoints were evaluated.

## 3. Results

### 3.1. Dataset Characteristics and Coding Agreement

A total of 20 minutes of synchronized video and sensor data were analyzed across four participants, corresponding to 240 five-second epochs. Across all epochs, percent agreement between behavioral coders was 89% with a Cohen’s K of 0.77. Participant-specific percent agreement between coders ranged from 85 to 97% (Table 2). Across all participants, the behavioral coding classified 37% of epochs as MVPA and 63% of epochs as SLPA. The median percentage of MVPA epochs per participant was 42.5% (range: 20–43%).

### 3.2. ROC Analysis and Group Cutpoint Determination

ROC analysis was performed using data from all four participants to determine an optimal activity count cutpoint distinguishing MVPA from SLPA epochs. The ROC curve demonstrated an AUC of 0.903, indicating activity count measures could discriminate between video coded MVPA and SLPA epochs across the range of possible cutpoints (Figure 3A). An optimal group cutpoint of 141 activity counts per epoch classified 46.25% of epochs as MVPA and 53.75% as SLPA. Using the group cutpoint, the overall percent agreement was 79% (Table 3). A depiction of the video-identified MVPA time and the sensor-identified MVPA time per participant, using the group cutpoint, is shown in Figure 3B.

### 3.3. Individual Cutpoint Analysis

Participant-specific ROC analyses were subsequently performed to determine individualized cutpoints for each toddler, with cutpoints ranging from 44–410 activity counts per epoch (Table 4). Application of individualized cutpoints improved overall accuracy in 3 out of 4 participants compared to the group-derived cutpoint. The average change in accuracy between the group-derived cutpoint and individualized cutpoints was +4.2% (range: −5% to +12%).

## 4. Discussion

This study established a method for quantifying MVPA and SLPA time in toddlers with DS participating in a home-based mobility intervention, using an accelerometer worn on the torso. The group-derived cutpoint demonstrated excellent classification performance (AUC = 0.903) with good sensitivity (94.4%), indicating that a single threshold can accurately identify periods of MVPA across children despite differences in individual movement patterns. Participant-specific cutpoints generally resulted in minimal improvements in classification. Though replication in a larger sample is warranted, these findings suggest that individualized calibration may not be necessary for classifying MVPA in toddlers with DS, making activity monitoring more practical for clinical interventions and larger research studies.

A primary contribution of this work is the validation of a group-derived cutpoint that demonstrated similar classification accuracy to individualized cutpoints. Individual calibration requires additional behavioral coding and cutpoint derivation for every participant, which is not practical in large samples. The use of a standardized cutpoint eliminates the need for repeated calibration procedures while maintaining excellent classification performance. This approach improves the feasibility of using wearable sensors to monitor activity in toddlers with DS and may facilitate implementation in clinical settings where individualized calibration is impractical.

Though the individual cutpoints performed similarly overall to the group cutpoint, the values themselves ranged widely. The cutpoint for one toddler (Participant 2) was much lower than the others; this participant was also the only one for whom the individual cutpoint had poorer percent agreement than the group cutpoint. This participant had the fewest true positive MVPA segments; given the greater asymmetry in data categorization, the Youden Index may not be the optimal measure to establish an individual cutpoint. Because toddlers with DS vary widely in their activity levels and mobility, we consider it a strength that our group cutpoint performs similarly for children with both lower and higher levels of physical activity. However, a larger sample size is needed to further evaluate performance across the range of activity levels.

Our findings are consistent with previous work establishing ActiGraph activity count cutpoints in young children. Trost et al. (2012) derived and cross-validated intensity-related ActiGraph cutpoints for TD toddlers using direct observation during structured play [19]. They reported excellent classification accuracy for moderate-to-vigorous physical activity (AUC = 0.90) using a cutpoint of 418 activity counts per 15-s epoch. When adjusted to our five-second epoch length, this cutpoint is approximately 139 counts per epoch, which is similar to the group-derived cutpoint of 141 counts per epoch in the present study. Despite the differences in movement context and task demands of the two studies, the similarity between these cutpoints suggests that a common activity-count cutpoint may distinguish gross MVPA across toddlers with and without DS. Future studies should evaluate this cutpoint with toddlers with and without Down syndrome under the same conditions to determine the reproducibility of this finding.

The high sensitivity observed in our study indicates that the group-derived cutpoint successfully identified nearly all periods of MVPA. However, specificity was comparatively lower (70.2%), suggesting that some SLPA epochs were classified as MVPA. This pattern indicates that our approach may favor identifying MVPA at the expense of additional false positives. One possible explanation is that the PUMA intervention encourages subtle postural adjustments and trunk movements that generate measurable activity counts despite being coded as SLPA during behavioral observation. These small torso shifts may be functionally meaningful during early mobility training but may not satisfy behavioral definitions of MVPA. Future work should further examine how different definitions of activity for behavioral video coding influence classification performance during intervention-based activities.

Wearable sensors are generally able to reliably distinguish MVPA from SLPA in children with and without various motor disabilities of all ages, from 0 months old through adolescence, though existing studies use a variety of sensor placements, number of sensors, and validation tasks [21,22,24,31,32]. Though disorder-specific cutpoints are widely used, this presents limitations in generalizability. Work specific to infants and toddlers with motor disabilities is more limited; however, emerging evidence suggests that sensors can discriminate between MVPA and SLPA in pre-ambulatory children with developmental disabilities. For example, Orlando et al. (2025) demonstrated that inertial sensor data could accurately distinguish MVPA and SLPA time in pre-ambulatory children with cerebral palsy using raw triaxial accelerometer signals [20].

An additional consideration when comparing these findings with the previous literature is sensor placement. To generate their cutpoints, Trost et al. (2012) positioned the ActiGraph against the child’s trunk at the level of the right hip using a vest during free play [19]. A systematic review of accelerometry to measure physical activity in infants and toddlers suggest that the waist is the most precise and most common placement; other studies cited placed sensors on the ankle or wrist [24]. Orlando et al. (2025), in their work with pre-ambulatory children with CP, used a single sensor positioned on the lateral thigh [20]. The present study collected movement data using one sensor integrated into the PUMA harness on the back of the trunk. Despite these differences in sensor configuration, the activity cutpoint identified in the present study closely matched previously established cutpoints derived from hip-mounted ActiGraph data.

This study has several limitations. First, the group-derived cutpoint was established using data from a relatively small sample of toddlers with DS. Although the cutpoint demonstrated consistent classification performance across participants and participant-specific cutpoints provided only minimal improvements, additional validation in a larger and more diverse cohort, including TD toddlers in matched contexts, is needed to confirm its generalizability. Second, the activity counts used in this study were derived from APDM Opal sensors using a preprocessing pipeline designed to reproduce ActiGraph activity counts, rather than measured directly from an ActiGraph device. Consequently, the cutpoint established in this study may not be directly transferable to other wearable sensors. Finally, the cutpoint was validated only during the PUMA home-based intervention while the sensor was worn on the torso. While our behavioral coding scheme was based on the previous literature [20] and aligns well with the sensor data, there may be circumstances of under or overreporting of active time as well as mismatch between the sensor readings and the behavioral coding due to the coding scheme definitions (e.g., leg movement without torso movement) and epoching of the data. The intervention provides postural and body-weight support, enabling toddlers to access postures and movement types that may differ from children’s typical physical activity throughout the day. Therefore, the cutpoint’s applicability to activities outside of the intervention is unknown. Future studies should validate this cutpoint in larger cohorts of toddlers with DS, across different wearable sensor systems, during different interventions and naturalistic play, and with children with variable activity levels. While this analysis was specifically designed to evaluate MVPA and SLPA time, future work could evaluate the extent to which wearable sensors can provide insight into the postures and/or types of locomotion engaged in by toddlers during the intervention.

Behavioral observation remains the standard for identifying MVPA and SLPA behavior; however, it requires extensive video recording, manual coding, and trained raters, making it both time-intensive and costly. Validating a standardized activity count cutpoint reduces the reliance on repeated behavioral coding for each participant while maintaining strong classification accuracy. This provides a practical approach for objectively monitoring activity during intervention sessions and may facilitate larger longitudinal studies evaluating changes in mobility over time.

## Figures and Tables

**Figure 1:**
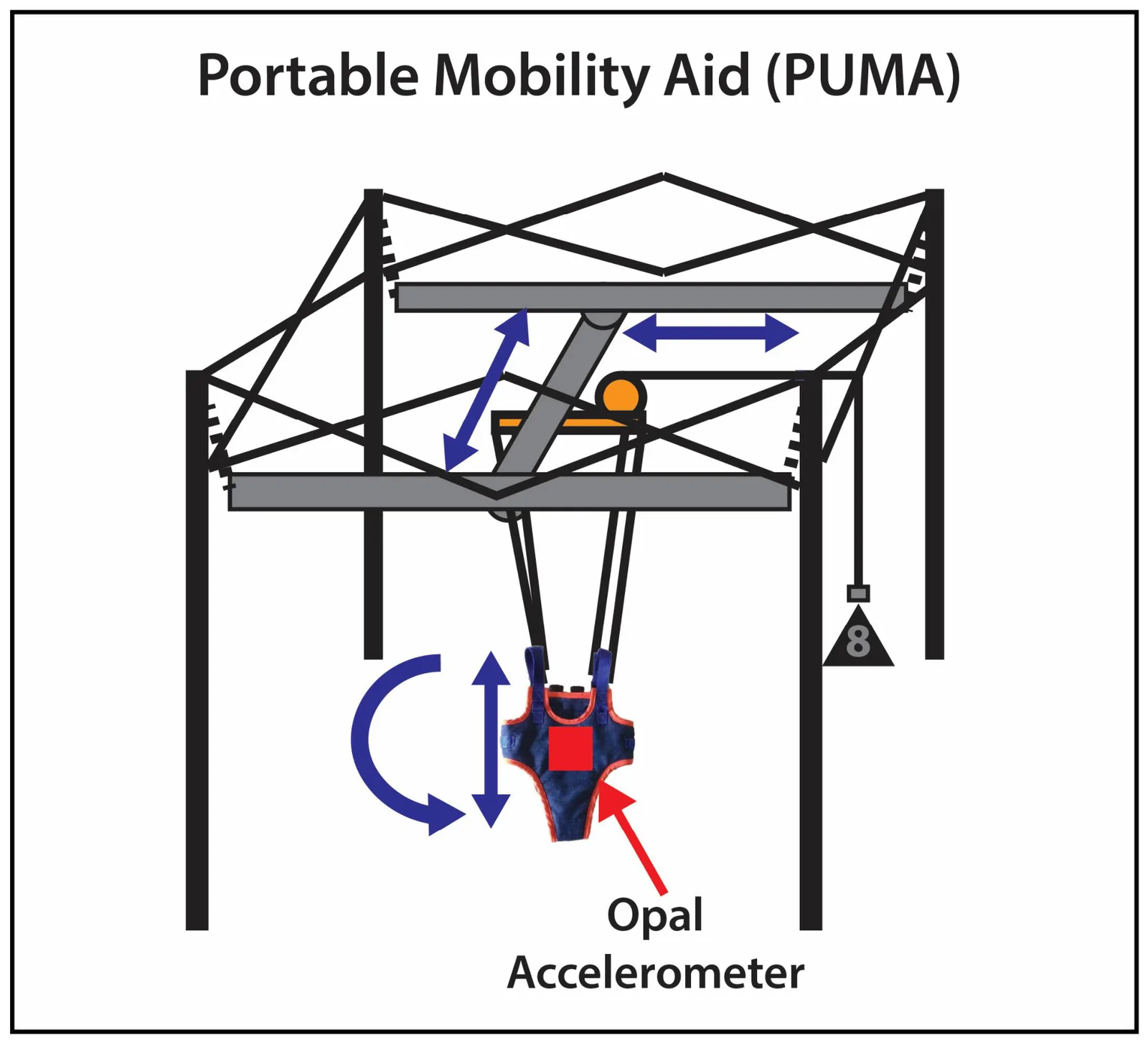
Schematic of the Portable Mobility Aid for Children (PUMA) body-weight support harness system. Children can move in any direction; the yellow bar slides along the gray bars left to right and front to back, and rotates with the child. The pulley system also allows the child to transition from seated to upright positions. The APDM Opal sensor was secured on the back of the onesie garment worn by the child and attached to the PUMA system. Adapted from Collimore-Doherty, A.N.; Sutter, E.N.; Wilkerson, E.; Baumer, N.T.; Iverson, J.M. Implementation and acceptability of PUMA Playtime intervention for toddlers with Down syndrome: a feasibility study. *Disabil Rehabil Assist Technol* **2026**, 1–12, doi:10.1080/17483107.2026.2716555 [28], with permission from the publisher (Taylor & Francis Ltd., https://www.tandfonline.com (accessed on 3 September 2026)).

**Figure 2:**
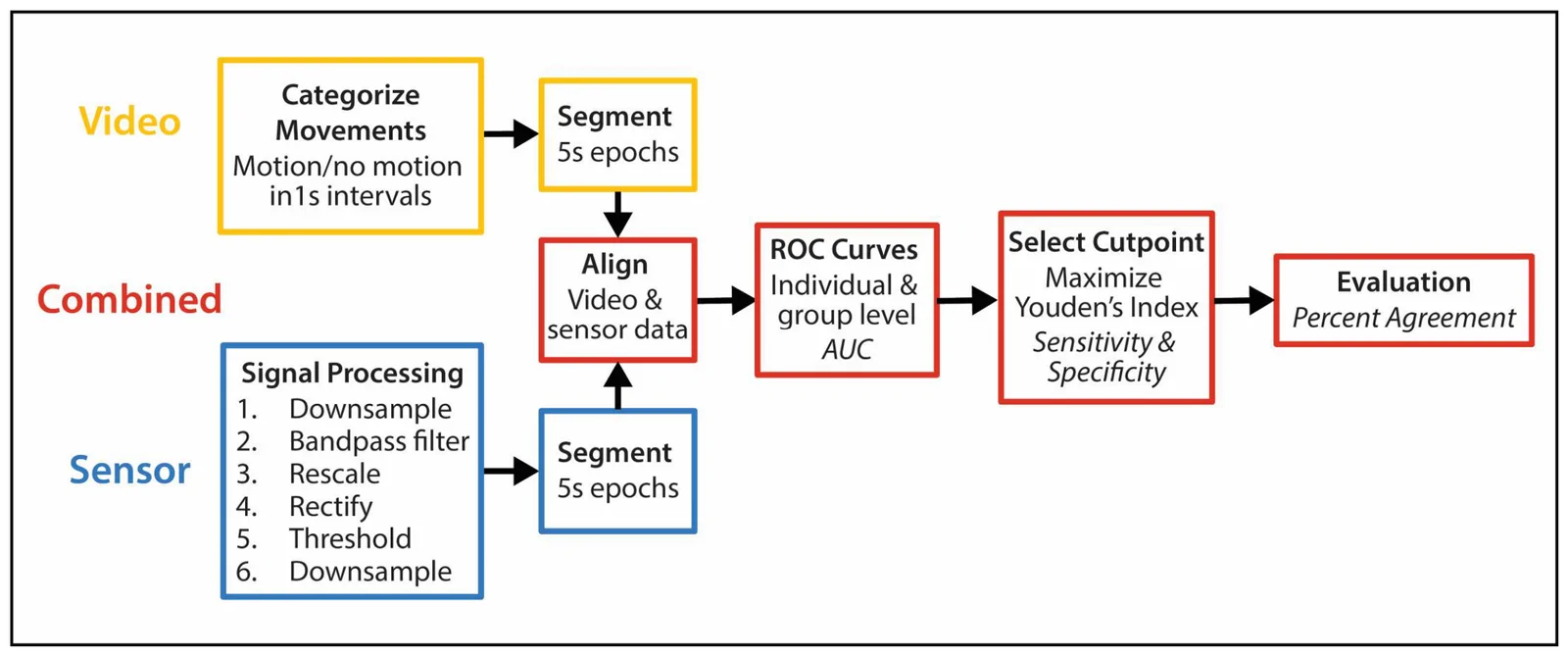
Workflow of all processing steps. Video and sensor data were analyzed separately and then aligned to calculate cutpoints and evaluate agreement. Evaluation metrics are italicized to indicate where in the workflow they were calculated. AUC = Area Under Curve.

**Figure 3:**
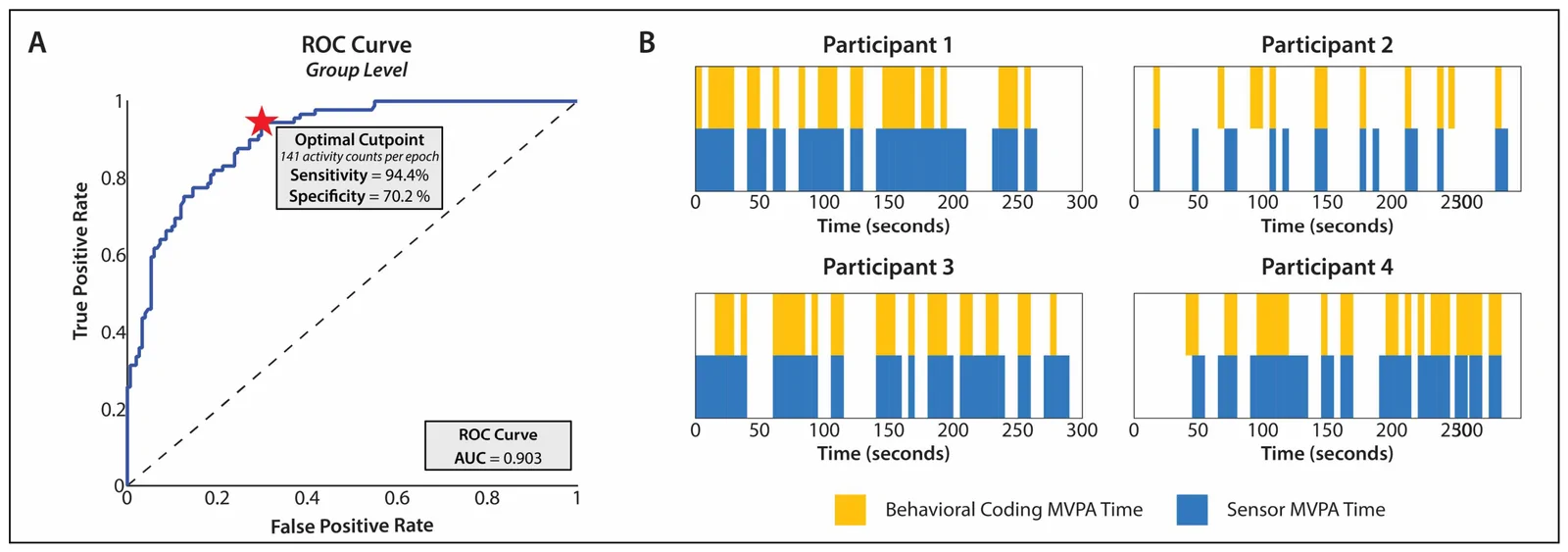
(**A**) Receiver Operating Characteristic (ROC) curve analysis used to determine optimal group cutpoint of 141 activity counts per epoch. The dashed line represents a non-discriminatory test. (**B**) Aligned MVPA time per participant as identified from behavioral video coding (yellow) and the processed wearable sensor data (blue) using the group cutpoint.

**Table 1:** Participant demographics.

Participant	Sex	Age (Months)	Race/Ethnicity
1	M	22.75	White/Not Hispanic or Latino
2	F	18.11	White/Not Hispanic or Latino
3	M	19.36	White/Not Hispanic or Latino
4	M	21.24	White/Not Hispanic or Latino

**Table 2:** Percent video-identified moderate-to-vigorous physical activity (MVPA) and sedentary-to-light physical activity (SLPA) time and video coding reliability.

Participant	Total Epochs	% MVPA	% SLPA	Coder Percent Agreement
1	60	43%	57%	93.33%
2	60	20%	80%	96.67%
3	60	43%	57%	81.67%
4	60	42%	58%	85%

**Table 3:** Performance of group-derived cutpoint.

Cutpoint	Agreement	Sensitivity	Specificity	False Positives	False Negatives	AUC
141	79.2%	94.4%	70.2%	29.8%	5.6%	0.903

**Table 4:** Individualized cutpoint performance by participant.

Participant	Individual Cutpoint	Individual Accuracy (%)	Group Accuracy (%)	Δ Accuracy (%)	Sensitivity	Specificity	AUC
1	305	88.3%	76.7%	11.7%	100%	58.8%	0.920
2	44	80%	85%	−5.0%	75%	87.5%	0.911
3	410	85%	78.3%	6.7%	100%	61.8%	0.900
4	319	80%	76.7%	3.3%	92%	65.7%	0.869

## Data Availability

The raw data supporting the conclusions of this article will be made available by the authors on request.

## Supplementary Materials

The following supporting information can be downloaded at: https://www.mdpi.com/article/10.3390/s26196002/s1, Supplementary Material S1: Behavioral Coding Protocol.

## Abbreviations

The following abbreviations are used in this manuscript:

DS	Down syndrome
TD	Typically developing
PUMA	Portable Mobility Aid for Children
ROC	Receiver operating characteristic
AUC	Area under curve
