# Supplementary material for: Quantifying Physical Activity in Toddlers with Down Syndrome with Wearable Sensors During a Home-Based Mobility Intervention
Source: Sensors (Basel). 2026 Sep 22;26(19):6002. doi: 10.3390/s26196002 (PMC13667628; doi:10.3390/s26196002)
Supplement: Supplementary file 1 [file sensors-26-06002-s001.zip › sensors-4481143-supplementary.pdf]

SUPPLEMENTARY MATERIAL S1
Behavioral Coding Protocol

1. Overview and General Guidelines
This behavioral coding scheme is designed to identify periods of toddler movement from video recordings. Motion included self-generated locomotion, transitions between postures, and substantial torso movement while in a singular posture.

- Motion was coded in 1-second intervals. If the toddler was moving according to the below guidelines for > 0.5 seconds of the interval, it was coded as "Motion."
- Caregiver-assisted movement was considered motion when the toddler's torso or pelvis was moving through space.
- Small movements such as fidgeting or wiggling that did not meet the criteria described below were not considered motion.

2. Coding Key

Code	Description
m	Motion
n	No Motion

3. Transitions Between Postures
Transitions between postures were considered motion and included transitions involving standing, sitting, squatting, kneeling, quadruped, prone, and supine positions. In general, motion onset was identified when the torso or pelvis started moving in the direction of the transition. Motion offset was identified when both the torso and pelvis remained stationary.

4. Locomotion
Locomotion was considered motion and included supported or unsupported walking, cruising, supported or unsupported knee walking, hands-and-knees or hands-and-feet crawling, army crawling, and bottom shuffling or hitching. Motion onset was identified when the torso, pelvis, or legs started moving in the direction of the locomotion. Motion offset was identified when the torso, pelvis, and legs remained stationary.

5. Movement Within a Single Posture
Movement occurring while the toddler remained within a single posture was coded as motion when one or more of the following criteria were met:

- Trunk movement visually estimated to be approximately 45° or greater in any direction.

- Knee movement visually estimated to be approximately 45° or greater while standing.
- Movement of the pelvis upward, downward, forward, backward, or laterally.

Examples: leaning forward or backward while sitting, kneeling, or standing; dancing or marching in place; pivoting; bridging while supine; and extending the trunk and extremities while prone.

The following movements were not coded as motion when they occurred without movement meeting the criteria above:

- Isolated arm movements.
- Isolated leg movements while sitting that did not result in qualifying trunk movement, such as kicking.
- Stepping in place when knee movement did not reach approximately 45°.
